# Upper Limb Design of an Anthropometric Crash Test Dummy for Low Impact Rates

**DOI:** 10.3390/polym12112641

**Published:** 2020-11-10

**Authors:** Marek Jaśkiewicz, Damian Frej, Dariusz Tarnapowicz, Milos Poliak

**Affiliations:** 1 Department of Automotive Engineering and Transport, Kielce University of Technology, 25-340 Kielce, Poland; 2 Faculty of Mechatronics and Electrical Engineering, Maritime University of Szczecin, 70-500 Szczecin, Poland; 3 Department of Road and Urban Transport, University of Žilina, 010-26 Žilina, Slovakia

**Keywords:** joint, dummy, collision biomechanics, accidents, road safety

## Abstract

The article presents the design of the upper limb joints of an anthropometric dummy intended for rear crash tests for low impact speeds. These joints represent the connection of the hand to the forearm, the forearm to the arm, and the arm to the shoulder. The designed joint is adapted to the construction of a dummy representing the 50th percentile male. The joints currently used on Hybrid III dummies require calibration after each crash test. The construction of the new joint ensures the appropriate strength of individual joint elements and the repeatable value of the joint characteristics without the need for frequent calibrations. The designed joints have the ability to set a variable stiffness characteristic, thanks to which it is possible to use this joint universally in dummies representing populations of other percentile sizes. The range of movement of the joints has been selected to reflect the range of mobility of the upper limb of an adult. The characteristics of the joints were compared with those used in the joints of the Hybrid III 50 percentile male dummy. Moreover, it should be noted that the constructed joints of the upper limb are made by hand; therefore, their comparison with the Hybrid III dummy shows some deviations in the moments of resistance. Making the joints with a 3D printer, taking into account the appropriate material, will ensure greater accuracy and will also result in joining the individual elements of the joint into a whole. The obtained results show slight differences between the moment of resistance in the joints of the constructed anthropometric dummy compared to the hybrid III dummy.

## 1. Introduction

**Figure 1:**
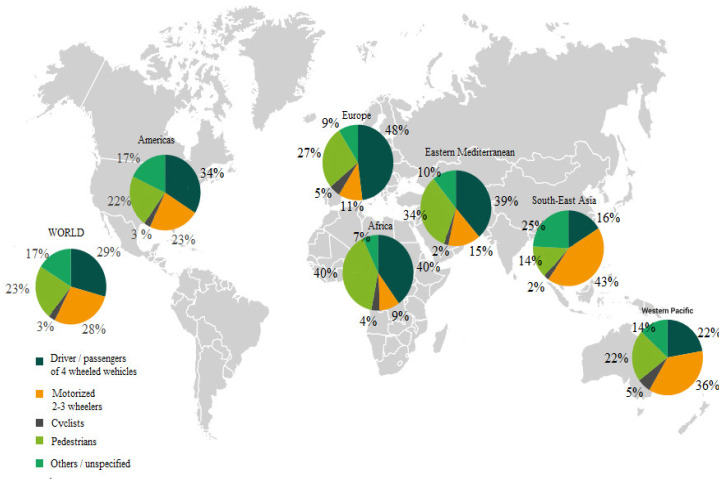
Distribution of fatalities in road accidents according to WHO by road user type [3].

Every year around the world, millions of people lose their lives or are seriously injured in road accidents. The World Health Organization (WHO) lists road accidents among the most common causes of death in the world [1]. In addition, there are more and more road fatalities in car accidents worldwide [2]. It is estimated that 1.2 million people worldwide die in road accidents each year, and as many as 50 million more are injured. This means that worldwide, more people die from road traffic injuries than from human immunodeficiency virus (HIV)/acquired immune deficiency syndrome (AIDS), tuberculosis or diarrheal disease [3]. Currently, road accidents are the most common cause of death among children and adolescents aged 5 to 29 worldwide. Forecasts indicate that road fatalities will be the fifth leading cause of death by 2030 [2,4]. In 2018, around 1.35 million people died on roads around the world. Europe is among the regions with the safest roads in the world [5]. The distribution of fatalities in road accidents according to WHO as a result of road accidents by type of road user is presented in Figure 1 [3].

**Figure 2:**
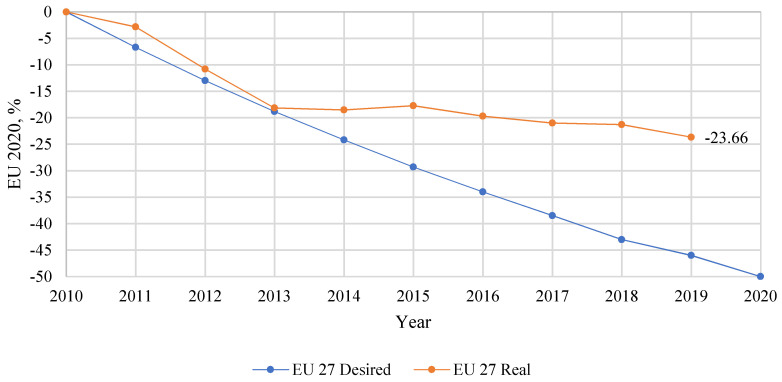
State between actual and desired progress towards the Europe 2020 target [11].

In 2018, the 28 Member States of the European Union reported around 25,100 killed in road accidents. The result is lower by 21% compared to the data from 2010 [5,6,7]. Strong and well-targeted efforts are now needed at all levels and in all sectors of the European Union. The initial plan to increase road safety in the European Union assumed a 50% reduction in road accidents by 2020 [8,9,10]. The progress of the plan implementation presented below shows that the assumptions of the policy of increasing road safety in the European Union significantly differ from the assumed plan (Figure 2). Over 10 years, the number of accidents was reduced by 23.66% [8,11,12].

**Figure 3:**
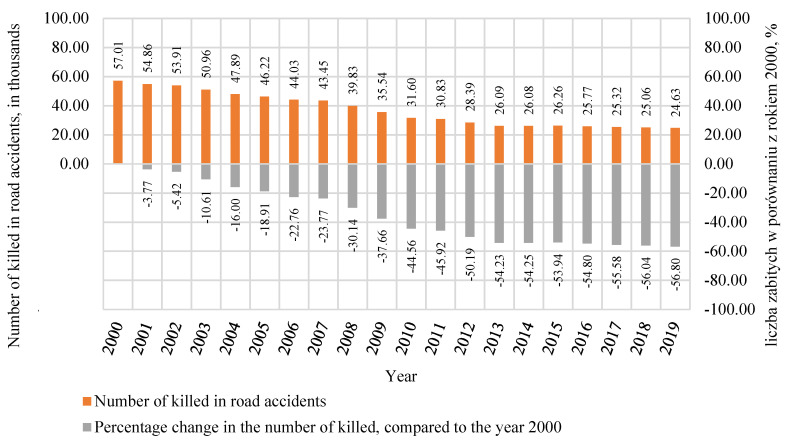
Number of fatalities in road accidents in Europe in 2000–2019 [11].

Thanks to continued social and technological development in Europe, the number killed in road accidents decreased by 56.8% between 2000 and 2019. Moreover, as many as 46% of those killed in road accidents are drivers and passengers of cars [8,9,10]. The number killed in road accidents on all roads in the European Union in the period from 2000 to 2019 [11] is shown in Figure 3. In total, 24,625 people died in road accidents in 2019 [2,11,13].

For the next decade, as part of the road safety policy for 2021–2030, the European Union has set itself a new target to reduce the number of fatalities in road accidents and the number of serious injuries by 50% by 2030 [2,3,11]. The Stockholm Declaration of February 2020 provides the basis for further global political commitments for the next decade. It is estimated that for every person killed in a road accident, there are another five who suffer serious injuries that cause a permanent change in the way of life (there were around 120,000 in 2019). The estimated external costs of road accidents amount to approx. EUR 280 billion, i.e., approx. 2 percent of European Union (EU) Gross domestic product (GDP) [14,15,16].

The Commission’s Strategic Road Safety Action Plan and its 2021–2030 policy also set out ambitious road safety plans to eliminate road fatalities by 2050 (“zero vision”). In order for the European Union to achieve “vision zero”, the European Commission is implementing a “secure system” throughout the EU. It includes safer vehicles, safer infrastructure, better use of protective equipment, lower speeds, and better post-accident care. In addition, the EU will work to improve cross-border enforcement of road traffic offenses, digitize driving licenses, and find new ways to help Member States with relatively underperforming road safety scores [17,18,19]. According to statistical data for 2019, in the European Union as many as 37% of all accidents occur in cities, while 8% occur on motorways, and 55% in rural areas. Accidents in urban areas are different in nature to accidents on rural roads and highways. First, in urban areas, pedestrians, not car passengers, account for the highest proportion of fatalities: in urban areas, 40% of road fatalities are pedestrians, 12% are cyclist, and 18% are two-wheelers. This means that 70% of all deaths in road accidents in urban areas are unprotected road users. Outside urban areas, this percentage is 34% [1,18].

The main indicator showing the level of road safety is the conversion of the number of people killed in road accidents per million inhabitants [20,21,22]. The European Union average in 2019 remained at 51 deaths per million inhabitants. The highest number of victims was in Romania (96 victims/million), Bulgaria (89 victims/million), and Poland (77 victims/million). Compared to 2018, when Poland ranked fifth, we were ahead of Croatia (73 victims/million) and Latvia (69 victims/million). In turn, the lowest deaths in road accidents per one million inhabitants were in Sweden (22 victims/million), Ireland (29 victims/million), and Malta (32 victims/million) [1,18]. On the basis of police statistics in Poland, the most common cause of road accidents caused by drivers is failure to respect the right of way and mismatching speed to traffic conditions. However, taking into account only city roads, the most common reason is not keeping a safe distance. Each vehicle driver is obliged to maintain the distance necessary to avoid a collision in the event of braking or stopping of the vehicle in front [1,11,21].

From the user’s point of view, the vehicle should be able to move efficiently while maintaining travel safety. Over the years, it has turned out that it is easier to increase the reliability of a car’s drive unit than to ensure the safety of the people traveling in it. The main problem to increase the safety of travelers and other road users is the lack of complete information on the behavior of the human body during a road accident. Most of the crash tests use anthropometric dummies [22,23]. These dummies can be divided into rear, side, and front crash test dummies [1]. Depending on the type of crash test, the dummies differ in their construction. Table 1 shows the compatibility level of selected dummies with the human body on a 10-point scale [18,24,25].

**Figure 4:**
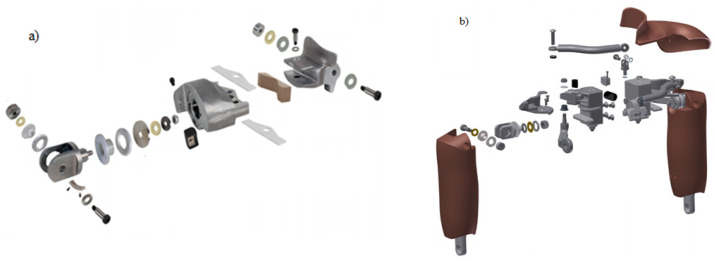
Shoulder joint used on a 50 percentile male dummy: (**a**) Hybrid III, (**b**) THOR [25].

Crash test dummies typically include a head assembly, spine assembly, thorax assembly, right and left arm assembly (including shoulder joint), and right and left leg assembly [19,25,26]. The right and left arm syndromes are divided into the upper and lower syndromes. The upper arm assembly is connected to the spine assembly of the anthropometric dummy [27,28]. The shoulder assembly should be designed to meet the anthropometric requirements for the specific percentile groups of the population of interest [25,29,30]. The individual dummy solids are connected by means of specialized joints. The disadvantage of these joints is the inability to adjust the connections without disassembling the dummy and the need to calibrate after each crash test [17,19,28]. Figure 4a shows the structure of the shoulder joint for the Hybrid III dummy representing a 50-percentile male, while Figure 4b shows the structure of the THOR dummy representing a 50 percentile male. The shoulder joint of the Hybrid III dummy consists of 18 elements; the joint is directly connected to the dummy’s spine assembly [27,28]. However, in the case of the THOR dummy, the structure of the shoulder joint is more complicated and consists of 30 elements [15,31,32].

An ideal design solution for anthropometric dummies intended for crash tests would be a design that would not require frequent calibration, while ensuring the durability of the joint and repeatability of results [15,25,31]. The THOR dummy is currently the most advanced crash test dummy. Its structure is similar to the human body; it has a developed spine with flexible joints in the thoracic and lumbar spine. General Engineering and Systems Analysis (GESAC) [25] developed the first THOR replacement model in 1995. The model was built to compete with the existing HYBRID III dummy model. In 2011, the administration of Highway Traffic Safety Nation commissioned the development and implementation of Humanetics’ men’s metric version of THOR—50 m, which is the 50th percentile of a human [24,25,28]. THOR—50M has better human body mapping and greater reproducibility of results [19,28,29]. It is characterized by better performance and durability than its competitor HYBRID III. The design advantage of THORA did not affect the popularity of the HYBRID III dummy because it is still the most used crash test dummy in the world. HYBRID III is used to evaluate the car’s safety systems in frontal impact tests. Both the THOR dummy and the HYBRID III dummy have an extensive network of sensors that collect information about the dummy’s behavior during a crash test [25,28,30].

There are still new concepts of interesting solutions for the construction of dummies similar to the human body. The need to build an anthropometric dummy for rear low-speed crash tests is a necessary aspect of increasing safety in road transport. Rear collisions are a fairly common type of accident [33,34]. In 2018, there were nearly 4000 of them, which corresponds to 12.6% of all accidents [34]. Compared to the total number of such events, they are relatively rarely tragic, accounting for 7.5 percent of all fatal accidents [25,35]. On the other hand, many participants in such accidents are injured. In the event of a rear-end collision, the passengers may face trauma to the cervical spine, which can cause painful complications that last for many years. These types of accidents often occur in built-up areas at low speeds [33]. However, they are most dangerous on the highway. When one car runs backwards into another at a speed of several dozen or more kilometers per hour, such a collision may end tragically [24,25,26]. Rear passengers are particularly at risk, and this is where children most often travel. The risk increases especially when the luggage compartment is relatively small and the passengers are close to the rear of the car. In addition, in many car models, access to the rear seats is more difficult than to the front seats. For this reason, emergency services can reach the victims later and provide help [25,36]. Rear impacts are the most common ones that occur in urban spaces. They happen both in traffic jams and during winter conditions, when the road surface is extremely slippery and braking is often impossible. Even at a relatively low speed, the driver has little room to maneuver on an icy road. In built-up areas, collisions may be the result of inattention, e.g., due to the use of a mobile phone while driving. Haste is also often to blame for example (e.g) when the driver accelerates, hoping to be able to pass the intersection before the traffic light turns red, and the car in front of him/her will stop. The most difficult, however, is to avoid rear-end collisions on a highway or motorway, where sudden braking of one vehicle may result in a collision [31,33,34].

A major problem in collecting information material during crash tests is the lack of a clear choice of a rear crash test dummy that replicates the movement of the dummy with the human body for low-impact velocities. One BioRID II (50th) anthropometric dummy is currently used for the rear crash tests, representing a 50th percentile male. For other percentiles of the human population, there are no rear crash test dummy versions [25,28,30]. Figure 1 shows the BioRID II (50th) dummy [25].

For crash tests, dummies are used that reflect the characteristics of the human body depending on age and weight. Dummies provide information about what happens in an accident with every part of a person [25,28]. Safety research is conducted by General Motors, which includes searching for new solutions and information regarding the safety of the driver, passengers, and children. The research carried out during crash tests on the mechanisms of injuries and responses to impacts, taking into account human tolerance, led to the creation of Hybrid III dummies. The Hybrid III dummies have a high index of differentiation with the human body. Research on dummies taking into account their similarities with the human body, led to the development of procedures for assessing head, face, chest, abdomen, and lower and upper limb injuries, which are used by all manufacturers around the world [15,28,36].

Shoulder joints used in anthropometric crash test dummies usually have to be disassembled, calibrated, and reinstalled after a series of collisions. In the case of a dummy representing the 50th percentile male or the 50th percentile female, the difference in the construction of the wrist will not be that marked, but in the case of the dummies representing children, the construction of the shoulder joint is different [24,25,37]. Dummies representing children 12 and 6 years old have a simple structure of a shoulder dummy that allows only the basic range of human body movement, while in the case of a dummy representing an 18-month-old child, this joint does not resemble the shoulder joint of the human body [25]. It is related to the mass of individual parts of the dummy’s body, which changes the stiffness characteristics of the shoulder joint. In the case of dummies used for lateral tests, the upper limbs are usually omitted in these tests; therefore, these dummies have a simple structure of shoulder joints [15,28,36].

The Hybrid III family of dummies is designed for leading crash tests while maintaining the speed of 30 km/h [22,27,38]. In addition, there are indications that the range of arm motion of the current frontal impact dummies may be a weak point in oblique and rear tests, where the dummies appear to slide out of the shoulder strap more easily than humans [25,39].

The lower impact velocities result in a different movement pattern of the dummy, which is mainly related to the current design of the joints. The design of the new joint has the ability to adjust the characteristics to the required one, which is optimal for the given conditions. Therefore, it is necessary to look for new design solutions that enable the best reproduction of human body behavior under specific conditions [22,27,38]

Undoubtedly, the use of 3D printers in modern times is becoming more and more popular [40,41]. In articles [42,43,44], 3D printers are used to construct prostheses of individual elements of the human body. It should be noted that the creation of prostheses with the use of 3D printing is cheaper and more easily available; the technologies create a specific type of prosthetic, These technologies create a specific type of prosthesis, for example a hand. Prostheses appearing on the market are more and more often focused on attracting customers with their appearance. In the works [45,46,47], 3D printing is used for the production of individual components of the robot hand. The manufactured elements are easily modifiable and constitute a significant part of the prototype elements, which are easy to manufacture with reduced costs. Moreover, it should be noted that 3D printing is not used for the production of anthropometric dummies, but the articles [48,49,50] present methods for increasing the accuracy of 3D printing. Undoubtedly, 3D printing offers great opportunities to build prototypes of complex steel devices that require complicated machining [51,52]. Therefore, it is worth considering the concepts of the construction of the entire joint mechanism used for the anthropometric dummy, because such a complicated structure would be accurately reflected and would enable a quick replacement of the entire joint with a new one.

## 2. Construction

**Figure 5:**
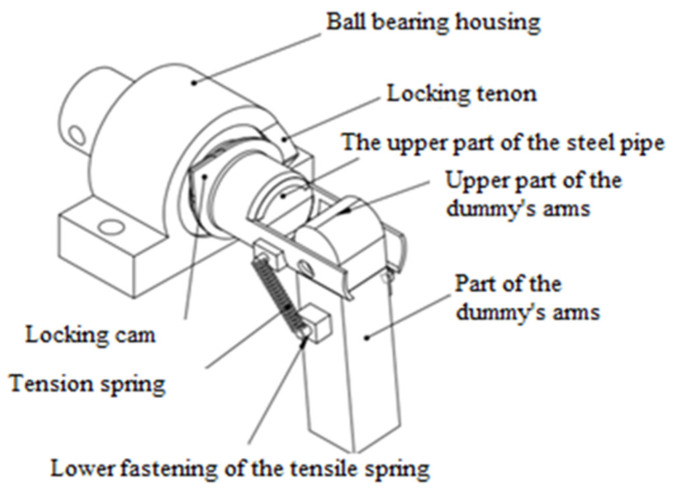
Construction of the shoulder joint of a crash test dummy.

**Figure 6:**
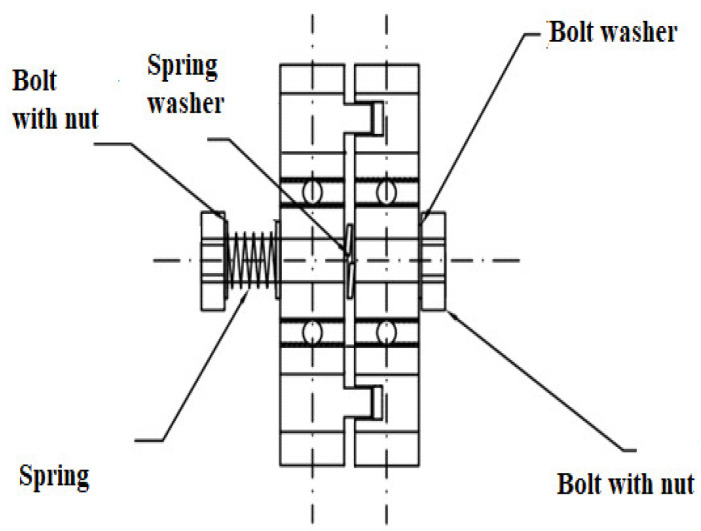
Construction of the elbow joint and wrist of a crash test dummy.

The innovative design solution of the upper limb enables the use of anthropometric dummies in each type, taking into account the population percentile measure. Figure 5 and Figure 6 show the joint of the shoulder joint and the elbow joint. Appropriately selected spring characteristics ensure obtaining the correct stiffness characteristics of the shoulder joint. It also provides an equal range of bend motion. The advantage of this design solution of the joint is that it is easy to replace the damaged element with a new one. In addition, the work of the joint does not require the need to disassemble the entire mechanism and calibrate the joint.

**Figure 7:**
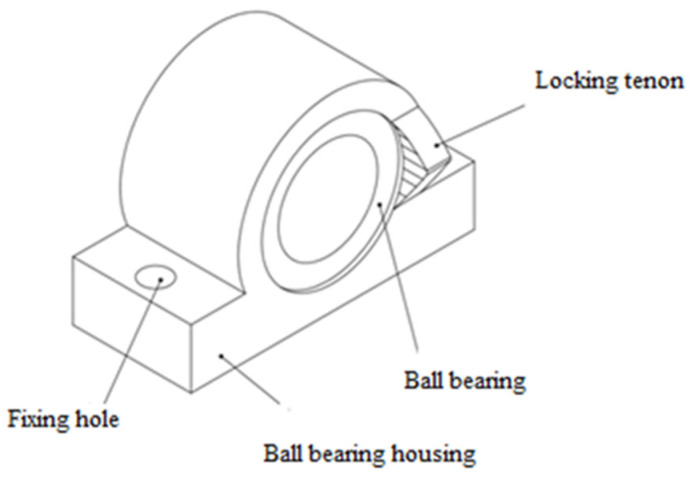
The first part of the shoulder joint.

**Figure 8:**
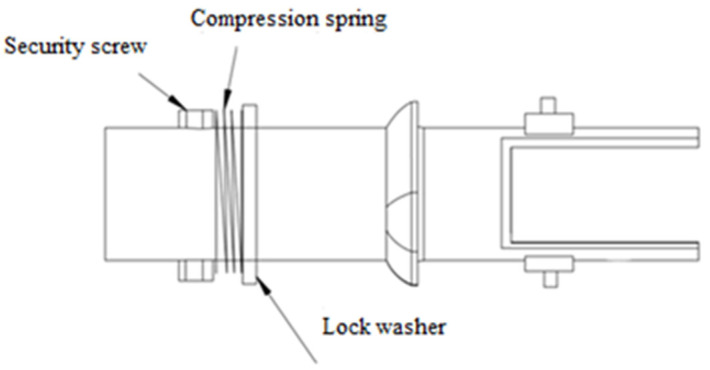
The second part of the shoulder joint.

The shoulder joint of the crash test dummy consists of three interconnected parts. The first part of the joint consists of a ball bearing which, in order to protect against mechanical damage, is mounted in the housing, and there is a locking tab on the housing. The first part of the joint is attached to the structure of the anthropometric manikin by means of two fixing holes. The first part of the articulation is shown in Figure 7. The second element of the dummy’s articulation consists of a steel pipe on which a locking cam has been mounted (Figure 8). The front side of the steel tube has cut holes for fixing the third joint element and the upper fixing of extension springs. In addition, in the rear part of the steel tube there is a securing hole in which a screw fits to prevent the steel tube from sliding out of the ball bearing. The third element of the shoulder joint is part of the arm of the crash test dummy. This element has holes for fixing the element to the steel pipe and lower fixing of extension springs.

When performing flexion and extension movements in the shoulder joint, the locking cam on the steel tube encounters a locking tab in the final stages of the movement, which prevents further movement.

Both the locking cam and the locking tab have 45° chamfered edges so that when the two elements touch, the joint movement is not immediately blocked, but the steel pipe extends from the ball bearing housing. Due to the pressure spring, the steel pipe cannot slide completely out of the ball bearing housing. The pressure spring is mounted on a steel tube in the rear part passing through the ball bearing housing. On the casing side, the spring is separated by a securing washer, while the protection against spring slipping on the other side is provided by a screw. If the steel pipe breaks out of the ball bearing housing, the pressure spring prevents the pipe from slipping out further, while preventing further movement in the joint. Due to the correct positioning of the locking tab and a properly selected spring, the joint has stiffness and elasticity characteristics similar to a human shoulder joint.

In the shoulder joint, abduction movements are possible thanks to the use of extension springs, which are attached to the steel tube in the upper position and attached to the arm element in the lower position. Together with the abduction movement, the springs extend until the upper part of the arm element meets the upper part of the steel tube. The arm element is connected to the steel pipe with a bolt. Proper selection of springs provides stiffness and elasticity characteristics similar to the human shoulder joint.

**Figure 9:**
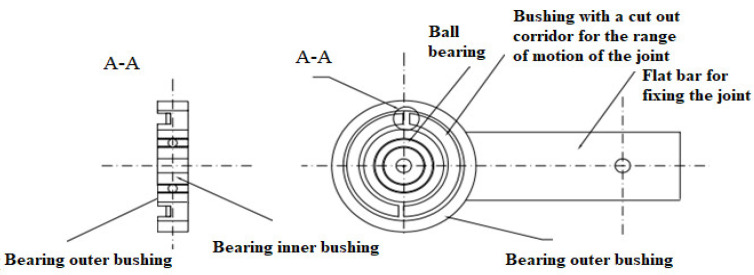
View of the left side of the elbow joint of the anthropometric crash test dummy.

**Figure 10:**
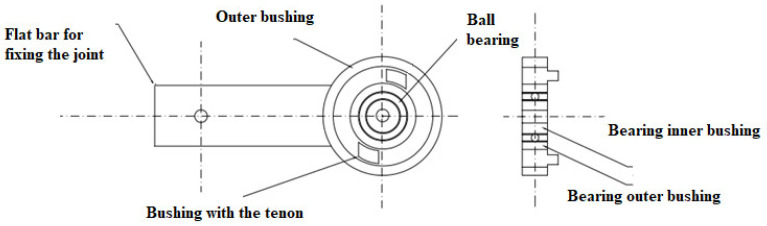
View right parts of the elbow joint of the anthropometric crash test dummy.

The elbow and wrist joints are equipped with two ball bearings, which form the right and left side of the joint. The bearings are protected by the inner sleeve of the ball bearing and the outer sleeve of the ball bearing. The right part of the joint has a sleeve with a burnt corridor of the joint’s range of motion, while the left part of the joint has a sleeve with a spline. The interconnected sleeves allow a range of motion of the joint suitable for the elbow joint of the human body. The joint range of motion of the corridor burnt onto the sleeve has a variable burn depth. Figure 9 shows a view of the left side of the elbow of the anthropometric crash test dummy, and in Figure 10, the right side of the elbow joint of the anthropometric crash test dummy is shown. The spline on the second sleeve moves freely inside the first sleeve burnout; when it encounters a change in the depth of the burnout at the end of the corridor, both parts of the joint repel each other. The right and left parts of the joint are separated from each other by a spring washer which is mounted to reduce the contact area of the joint parts. The spring washer facilitates mutual pushing off of the joint parts. The joint is connected by a bolt with a nut. The joint can be adjusted by a spring mounted on a bolt with a nut. The pivoting parts of the joint face resistance from the spring which prevents the joint parts from pushing against each other further. The joint can be adjusted by a spring mounted on the bolt. By setting the appropriate screw tightening torque, the joint has a stiffness and elasticity characteristic similar to that of a human elbow joint. The screw is separated from the spring by a spring washer, thanks to which the spring remains in a fixed position without changing its position during the movement of the joint.

**Figure 11:**
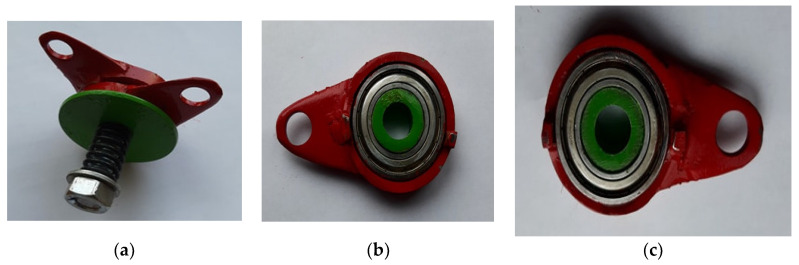
Designed elbow joint of the anthropometric dummy: (**a**) view of the entire joint, (**b**) view of the right part of the joint, (**c**) view of the left part of the joint.

The advantage of this design solution of the elbow joint is the easy replacement of the damaged element with a new one, without the need to disassemble the entire mechanism. This joint has an adjustable joint friction joint, so that this type of solution can also be used in the knee and wrist joints of a crash test dummy body. Patent applications have been filed for the above solutions [40,41]. Figure 11 shows the elbow joint made.

## 3. Results

The shoulder joint of the dummy being built was compared to that of the Hybrid III 50 percentile male. The maximum angle of the range of motion in the joint for flexing and extending the arm was measured using a protractor positioned at the center of the joint axis. The comparison of the maximum angle for bending and straightening of the constructed shoulder joint with the Hybrid III dummy is presented in Table 2.

**Figure 12:**
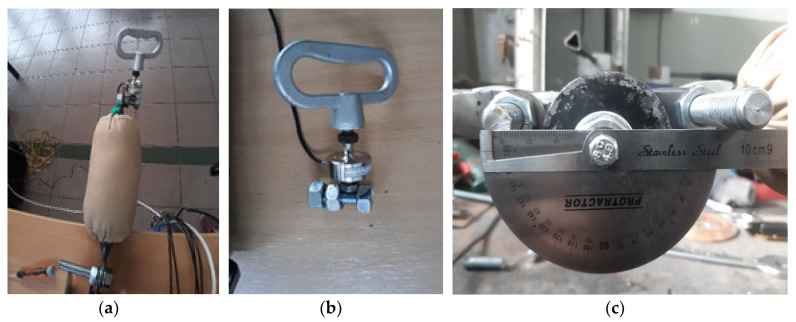
Measurements of basic parameters in the designed joint: (**a**) measurement of the resistance moment in the joint, (**b**) strain gauge for measuring the force in the joint, (**c**) measurement of the angle in the joint.

The stiffness characteristics were assessed on the basis of the point results. Measurements for the individual joints were made with a strain gauge force sensor (Figure 12b), while measurements of the range of motion of individual joints were measured with a protractor (Figure 12c). The measurement of the moment of resistance in the elbow joint is shown in Figure 12a. Based on the research results, the moment of shoulder joint resistance was determined. The moments of resistance in individual joints were determined using Equation (1)

(1) $$M=M_{k}\left(\Delta \varphi \right)+M_{c}\left(\Delta \dot{\varphi }\right)+C$$

where:

$M_{k}\left(\Delta \varphi \right)$—component being a function of the joint articular,
$M_{c}\left(\Delta \dot{\varphi }\right)$—component being a function of angular velocity in the joint,
$C=M_{o}-M_{k}\left(\Delta \varphi_{o}\right)$—fixed value selected on the basis of initial conditions,
$M_{0},\;\Delta \varphi_{o}$—moments and initial joint angles determined on the basis of geometry and equilibrium conditions of the dummy.

**Figure 13:**
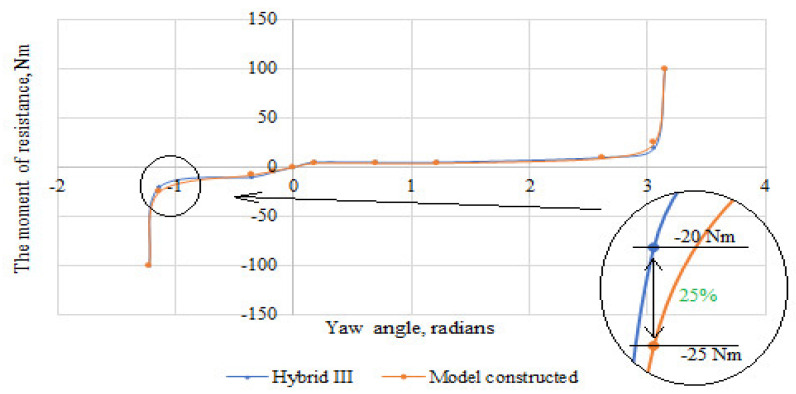
Summary of the stiffness characteristics of the shoulder joint of the constructed anthropometric dummy with the Hybrid III dummy.

**Figure 14:**
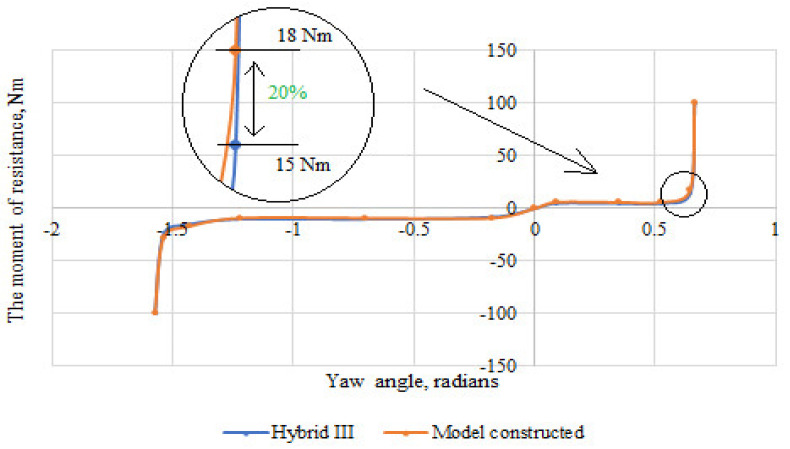
Summary of the stiffness characteristics of the elbow joint of the constructed anthropometric dummy with the Hybrid III dummy.

**Figure 15:**
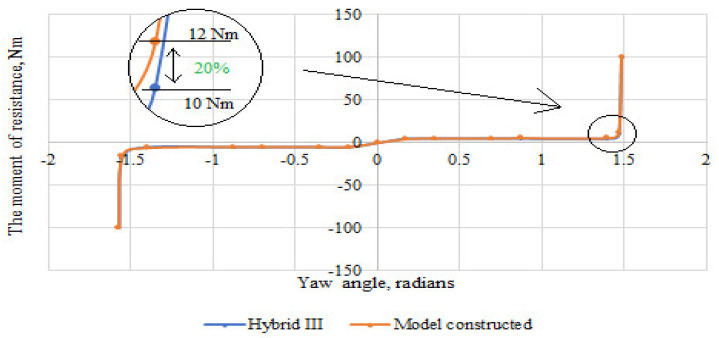
Summary of the stiffness characteristics of the wrist joint of the constructed anthropometric dummy with the Hybrid III dummy.

Based on the scores, the stiffness characteristics of the shoulder, elbow, and wrist joints were developed. The obtained characteristics were compared with those of the joint stiffness of the Hybrid III dummy representing a 50 percentile male. The difference between the moment of resistance in the upper limb joints of the constructed dummy in relation to the joints of the Hybrid III dummy is shown in Figure 13, Figure 14 and Figure 15. The greatest difference between the moment of resistance in the shoulder joint of the constructed dummy and the hybrid III dummy occurs at a deflection angle of −1.13 radians. The value of the moment of resistance at this point for the constructed dummy is 25 Nm and for the Hybrid III dummy it is 20 Nm; therefore the difference is 25%. In the case of the elbow joint, the greatest difference is seen at a deflection angle of 0.65 radians. The moment of resistance at this point is 15 Nm for the hybrid III and 18 Nm for the manikin under construction, so the difference is 20%. In contrast, for the wrist joint, the greatest difference occurs at a deviation angle of 1.47 radians. The moment of resistance at this point is 10 Nm for the hybrid III and 12 Nm for the manikin under construction, so the difference is 20%.

## 4. Conclusions

For crash tests, dummies imitating the features of the human body in terms of age and weight are used. There are dummies for frontal, side, and rear impact tests. Dummies provide information on what happens to different parts of the human body at the time of an accident. The currently used crash test dummies are a structure that records data from more than 200 sensors located in the dummy’s skeleton. This greatly affects the price of the dummy.

The currently used joints connecting individual body parts of anthropometric dummies should be disassembled and recalibrated after each crash test. In addition, their choice is closely related to the weight of individual body parts.

The designed upper limb is characterized by a simple structure that enables easy repair or replacement of a damaged joint element with a new one. Moreover, the design of the joint does not require unscrewing the entire mechanism and calibration of the joint. The versatility of the use of the checked joints allows them to be installed in the remaining percentiles of anthropometric dummies. In addition, the elbow joint can be used with the appropriate setting of the range of motion as a wrist or knee joint.

The upper limb constructions used in anthropometric dummies differ between the male percentiles and between the female and male dummies. It can be concluded that almost every dummy intended for crash tests has an individual design. The exceptions are joints used in dummies representing 3-year-old children, which more closely resemble the design solutions of a doll than other anthropometric dummies. The constructed joints were subjected to a series of 50 measurements in order to estimate the degree of component wear and potential deviations from values. During these tests, the same angular deflections and moments of resistance were recorded.

The joints used in the upper limb assembly are characterized by the fact that they can be used in any anthropometric dummy intended for rear crash tests, and the simple design allows for easy calibration of the joints in order to obtain the appropriate range of stiffness for individual population percentiles.

The process of building an anthropometric dummy is a complex stage in which the testing of individual joints and body parts plays an important role. In further tests, the moment of resistance in the individual joints will be measured before and after a controlled crash test at low speed.

In the history of crash tests, human corpses were the first dummies. Then, the first dummies were built by combining wooden elements with steel elements. Today’s dummies are very advanced, but they still require greater compatibility with the human body. As part of increasing the accuracy of the execution of individual elements of the upper limb, in further tests, the joints will be made using a 3D printer with appropriately selected materials. Perhaps in the future, 3D printers will allow the production of individual parts of the dummy’s body [53,54] or perhaps the entire dummy for crash tests [55].

## Figures and Tables

**Table 1:** Bio-fidelity comparison of side impact dummies [18,24,25].

Body Parts	World SID 50th	BioSID	EuroSID-2	EuroSID-1
Head	10.0	6.7	5.0	5.0
Neck	5.2	6.7	4.4	7.8
Shoulder	7.0	7.3	5.3	7.3
Thorax	7.9	6.3	5.2	5.4
Abdomen	6.4	3.8	2.6	0.9
Pelvis	7.8	4.0	5.3	1.5
Overall	7.3	5.7	4.6	4.4

**Table 2:** Maximum value of the rotation joint of the anthropometric dummy and the Hybrid III dummy representing the 50th percentile male.

Section	Construction of a Dummy		Hybrid III	
	∆φ_min_ [deg]	∆φ_max_ [deg]	∆φ_min_ [deg]	∆φ_max_ [deg]
Shoulder joint	70	180	70	180
Elbow joint	0	150	0	150
Wrist joint	90	85	90	85
